# Nonlinear Optics in Dielectric Guided-Mode Resonant Structures and Resonant Metasurfaces

**DOI:** 10.3390/mi11040449

**Published:** 2020-04-24

**Authors:** Varun Raghunathan, Jayanta Deka, Sruti Menon, Rabindra Biswas, Lal Krishna A.S

**Affiliations:** ECE Department, Indian Institute of Science, Bangalore 560012, India; deka@iisc.ac.in (J.D.); sruti@iisc.ac.in (S.M.); rabindrab@iisc.ac.in (R.B.); lalkrishna@iisc.ac.in (L.K.A.S.)

**Keywords:** optical resonances, nonlinear optics, harmonic generation, four-wave mixing, optical switching, sub-wavelength gratings, Mie scattering, Fano resonances, guided-mode resonance

## Abstract

Nonlinear optics is an important area of photonics research for realizing active optical functionalities such as light emission, frequency conversion, and ultrafast optical switching for applications in optical communication, material processing, precision measurements, spectroscopic sensing and label-free biological imaging. An emerging topic in nonlinear optics research is to realize high efficiency optical functionalities in ultra-small, sub-wavelength length scale structures by leveraging interesting optical resonances in surface relief metasurfaces. Such artificial surfaces can be engineered to support high quality factor resonances for enhanced nonlinear optical interaction by leveraging interesting physical mechanisms. The aim of this review article is to give an overview of the emerging field of nonlinear optics in dielectric based sub-wavelength periodic structures to realize efficient harmonic generators, wavelength mixers, optical switches etc. Dielectric metasurfaces support the realization of high quality-factor resonances with electric field concentrated either inside or in the vicinity of the dielectric media, while at the same time operate at high optical intensities without damage. The periodic dielectric structures considered here are broadly classified into guided-mode resonant structures and resonant metasurfaces. The basic physical mechanisms behind guided-mode resonances, electromagnetically-induced transparency like resonances and bound-states in continuum resonances in periodic photonic structures are discussed. Various nonlinear optical processes studied in such structures with example implementations are also reviewed. Finally, some future directions of interest in terms of realizing large-area metasurfaces, techniques for enhancing the efficiency of the nonlinear processes, heterogenous integration, and extension to non-conventional wavelength ranges in the ultra-violet and infrared region are discussed.

## 1. Introduction

The field of nonlinear optics encompasses the study of the nonlinear interaction of incident light with material at sufficiently high optical intensity levels resulting in the generation of harmonics, conversion of frequencies from one band to another, ultra-fast switching etc. [1,2,3]. The materials of interest for realizing nonlinear optical devices are typically in bulk form with interaction lengths in the millimeter to even kilometer range to allow efficient build-up of the nonlinear signal of interest. With the emergence of accurate nanofabrication techniques, there is interest in exploring nonlinear optical effects within the purview of nano-photonics with structural dimensions comparable to or much less than the incident light wavelength [4]. At such length scales, interesting regimes for studying nonlinear optics emerge, in which the resonant optical interaction due to frequency selective light scattering or frequency selective light coupling into and out of the structures becomes significant [5]. The resonant effects lead to a build-up of electric field inside or in the vicinity of the structure, resulting in enhancement of the nonlinear optical effects being studied. The structures of interest for such studies can be broadly divided into metallic structures which support plasmonic-type resonances, [6] and dielectric structures which support Mie scattering-type resonances [7]. Plasmonic sub-wavelength structures support field localization at its periphery due to localized surface-plasmon resonances with multipolar electric-type characteristics. This can be contrasted with dielectric structures which support both electric- and magnetic-type resonances with field concentrated either inside or outside the structure. The plasmonic resonances generally result in higher field enhancement when compared to the dielectric resonances, however at the price of higher absorption losses due to free-carrier absorption and reduced optical damage thresholds. Plasmonic structures have been extensively explored for nonlinear optical studies over the past decades, with many good review articles written in this topic [8,9,10]. In recent times, there has been a resurgence of interest in studying dielectric periodic structures, with the focus on realizing low-loss, sub-wavelength artificially engineered surfaces, also popularly called as metasurfaces for shaping the amplitude, phase and polarization properties of light to realize ultra-thin lenses, polarizers, holograms etc. [11,12,13]. This has also triggered interest in artificially engineered dielectric surfaces consisting of one or two- dimensional grating structures for resonant nonlinear optical studies [14]. In this context, high refractive index materials are particularly explored due to the enhanced field concentration which can be achieved when compared to medium- or low-index materials. Materials such as Silicon, Germanium, and Gallium Arsenide which possess high refractive indices are also amenable to large area nanofabrication due to their existing use in complementary metal-oxide semiconductor (CMOS) compatible microelectronics and integrated optoelectronic applications [15,16]. There is also interest in exploring interesting physical mechanisms to create the optical resonances in such structures, for example using guided-mode resonances [17], electromagnetically-induced transparency (EIT) like resonances [18] and bound-states in continuum resonances [19] to enhance local electric fields and consequently amplify the nonlinear optical effects.

This review article is aimed at discussing some of the recent research efforts pursued in the study of resonant nonlinear optical effects in high-index contrast dielectric-based arrayed structures. First, a comparison of light scattering from isolated dielectric particles with that of resonant transmission or reflection spectra obtained with arrays of such dielectric elements in the form of one-dimensional and two-dimensional grating-like structures is presented. The benefits of using high-index dielectric particles when compared to low- or medium-index particles in terms of the robustness of the scattering features are discussed. Various physical mechanisms responsible for the resonant features observed in periodically arranged sub-wavelength dielectric structures are also discussed. In this context, guided-mode resonance phenomenon due to frequency selective in- and out-coupling of the incident light into transverse propagating waveguide modes of the high index structure are discussed. Guided-mode resonance structures in the form of fully-etched high contrast gratings, and partially-etched zero contrast gratings are studied. EIT-like resonance phenomenon due to the coupling of bright excitable modes with dark unexcitable modes is discussed. The recent research into bound-state in continuum which leads to discrete resonant states in a continuum of states to realize high quality factor resonances using asymmetric structures or off-axis excitation is also briefly discussed. Subsequently, various nonlinear optical phenomena and structures studied in dielectric metasurface platform are discussed. This includes second- and third- harmonic generation processes, wave-mixing processes in the form of sum-frequency and four-wave mixing, higher harmonic generation processes, ultra-fast optical switching utilizing electronic nonlinearities, photon acceleration processes in harmonic generation, and hybrid metasurfaces for nonlinear optical applications. Finally, the main conclusions of this review article with some future directions of interest in terms of large-area metasurfaces, techniques for enhancing the efficiency of the nonlinear processes, heterogenous integration, and extension to non-conventional wavelength ranges in the ultra-violet and infrared region are presented. There are few other detailed review articles published previously in the area of all-dielectric metasurfaces [12,13], resonant grating structures [17], and their nonlinear optical application [4,10,14,20].

## 2. Design Considerations for Resonant Dielectric Grating Structures 

In this section, the resonant enhancement of the incident field in the dielectric sub-wavelength structures, which can be designed with an understanding of how the electromagnetic wave interacts with isolated entities and arrays of repetitive units is discussed. Furthermore, the role of refractive index of the dielectric structure in determining the extent of field enhancement within the structure is discussed. High index materials such as silicon and germanium are found to particularly result in well defined, high quality factor resonant spectral features.

### 2.1. Isolated Particle Versus Array

**Figure 1:**
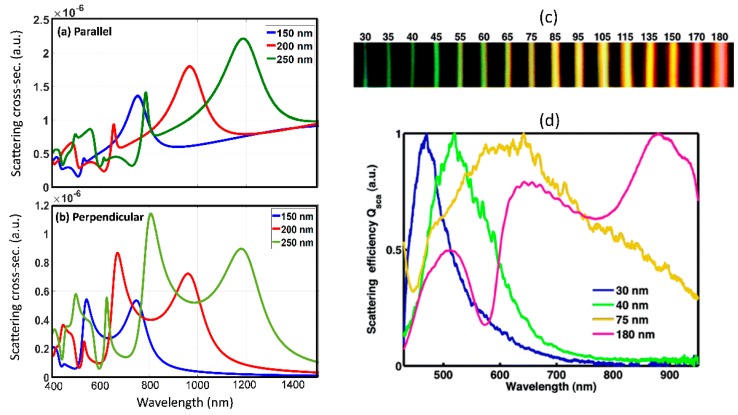
The simulated scattering cross section (in arbitrary units–a.u.) for silicon nanowires of varying diameter with incident light polarization oriented: (**a**) parallel and (**b**) perpendicular to the nanowire. (**c**) Experimentally obtained dark field images of nanowires showing light scattering for various width. (**d**) Experimentally obtained scattering spectra of nanowires of varying width. (Figures c and d are reproduced with permission from ref. [31]).

**Figure 2:**
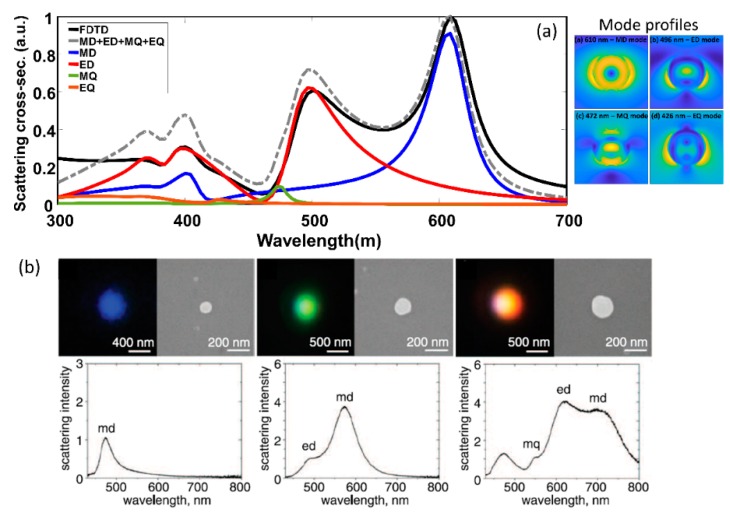
(**a**) Simulated scattering cross section (in arbitrary units–a.u.) for silicon nanospheres of fixed diameter of 150 nm (shown in black) and the result of decomposing the scattering spectra into magnetic dipole—MD (blue), electric dipole—ED (red), magnetic quadrapole—MQ (green) and electric quadrapole—EQ (brown). The sum of the MD, ED, MQ and EQ spectra is also shown (grey dashed). The field profiles for the MD, ED, MQ and EQ modes are also shown. (**b**) Experimentally obtained dark field scattering images and spectra for varying diameters of silicon sub-wavelength nanoparticles. (Figure b reproduced with permission from ref. [33]).

The interaction of electromagnetic fields with isolated sub-wavelength particles results in interesting linear light scattering which can be decomposed into basic electric and magnetic scattering modes. Plasmonic structures which rely on isolated, oligomeric and periodic array arrangement of metal sub-wavelength sized particles have been studied extensively over the last five decades [21]. Such structures rely on localized surface-plasmon resonances for field enhancement and find applications in fluorescence [22,23], Raman scattering [24,25,26], and nonlinear optical studies [8,9,10]. Plasmonic resonances result in multi-fold field enhancement with only electric multipolar characteristics and field localization outside the structure in order to satisfy the required boundary conditions. In similar lines to light scattering from metallic nanostructure, size-dependent resonant light scattering from dielectric nanostructures has also been studied [27]. The field enhancement in dielectric sub-wavelength structures is not as large as in plasmonic structures, however the field concentration inside the dielectric, the high damage thresholds and the robust scattering spectra has triggered interest in studying light scattering from dielectric particles in isolated and array form [28]. Figure 1a,b shows the simulated scattering spectrum from an isolated silicon cylindrical nanowire with varying diameter. Such isolated wires can be grown using chemical synthesis techniques and suspended in solution form [29] or can also be defined using nano-lithography techniques [30]. Experimentally reported study of scattering spectra from silicon nanowires of varying widths is also shown in Figure 1c (from [31]). In this study, the size-dependent visible resonant scattering from the nanowires results in vivid colors obtained using dark-field imaging. The experimental and simulated scattering spectra in Figure 1 are found to be in good agreement. Figure 2a shows the simulated scattering cross-section from an isolated silicon nanosphere of 150 nm diameter, which is further decomposed into characteristic electric and magnetic resonances. The scattering spectra for a sub-wavelength spherical particle, expressed as scattering efficiency can be expanded as a superposition of characteristic electric and magnetic resonant modes as follows: [7]

(1) $$Q_{scatter}=\frac{2}{x^{2}}\overset{\infty }{\underset{i=1}{\sum }}\left(2i+1\right)\left({\left|a_{i}\right|}^{2}+{\left|b_{i}\right|}^{2}\right)$$

(2) $$where,\;a_{i}=\frac{m^{2}j_{i}\left(mx\right){\left[xj_{i}\left(x\right)\right]}^{\prime }-\mu_{1}j_{i}\left(x\right){\left[mxj_{i}\left(mx\right)\right]}^{\prime }}{m^{2}j_{i}\left(mx\right){\left[xh_{i}\left(x\right)\right]}^{\prime }-\mu_{1}h_{i}\left(x\right){\left[mxj_{i}\left(mx\right)\right]}^{\prime }}$$

(3) $$\;b_{i}=\frac{\mu_{1}j_{i}\left(mx\right){\left[xj_{i}\left(x\right)\right]}^{\prime }-j_{i}\left(x\right){\left[mxj_{i}\left(mx\right)\right]}^{\prime }}{\mu_{1}j_{i}\left(mx\right){\left[xh_{i}\left(x\right)\right]}^{\prime }-h_{i}\left(x\right){\left[mxj_{i}\left(mx\right)\right]}^{\prime }}$$

where *a_i_*, *b_i_* are the electric and magnetic mode coefficients respectively, which are expanded in terms of Bessel, Hankel, Ricatti-Bessel and Ricatti-Hankel functions, *x* = k*a* refers to the modified dimension parameter, and $m=\frac{\sqrt{\varepsilon_{1}\mu_{1}}}{\sqrt{\varepsilon_{host}\mu_{host}}}$ refers to the contrast parameter [7]. Simplified forms of the scattering expansion for specific structures can be found in ref. [32]. Typical field profile obtained close to the electric/magnetic dipolar and quadrapolar resonances for isolated spherical particle are also shown in Figure 2a.

**Figure 3:**
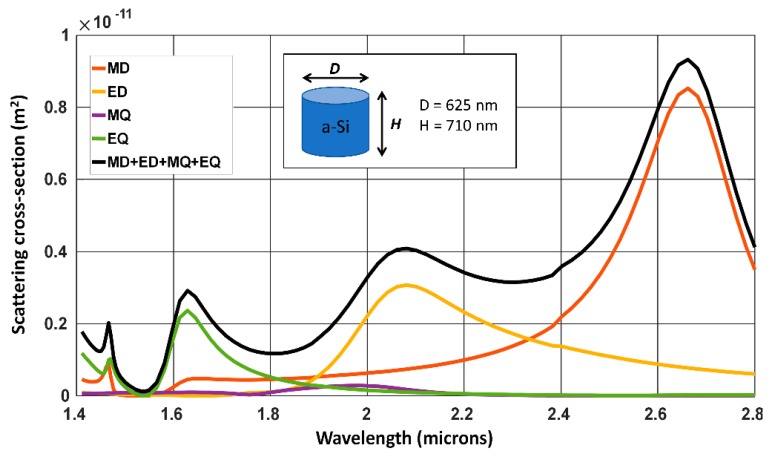
Scattering spectra from an isolated sub-wavelength cylinder separated into: magnetic dipole —MD (red), electric dipole—ED (orange), magnetic quadrapole—MQ (purple) and electric quadrapole—EQ (green). The sum of the MD, ED, MQ and EQ scattering spectra is shown in black.

The experimental demonstration of tunability of the scattering spectrum based on dielectric particle size is shown in Figure 2b (from [33]). For certain particle diameter, strong influence from magnetic dipole mode is observed (denoted by md in Figure 2b). The study of magnetic resonances in dielectric structures, in particular magnetic dipole modes has been of particular interest for the resonant enhancement of nonlinear optical effects [34] and can potentially be used to enhance light-matter interaction in materials with allowed magnetic transitions [35]. Figure 3 shows the scattering spectra for a silicon isolated sub-wavelength disk. The scattering spectra from sub-wavelength dielectric disks resemble that of sub-wavelength spheres with analytical models available for decomposition into magnetic and electric modes. Sub-wavelength disks are structures which can be fabricated using standard electron-beam lithography and etching processes, and are best suited for large areas, reproducible scaling for practical photonic device applications [36]. These are often studied in isolated, closely spaced arrays, and in collective oligomeric forms [37].

**Figure 4:**
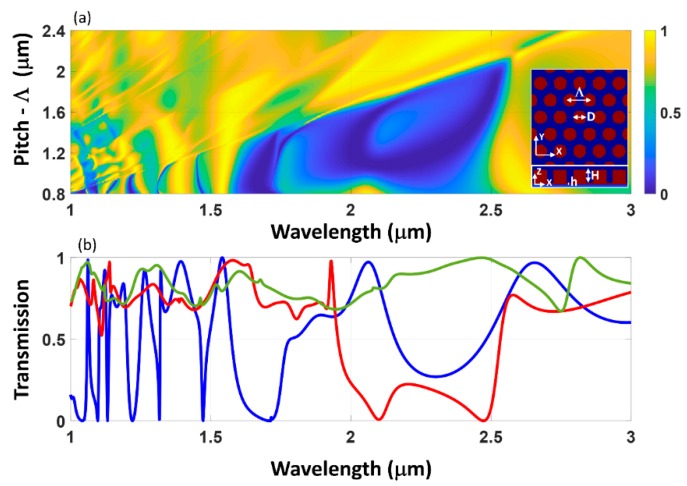
(**a**) Transmission spectra contour map for varying array pitch (Λ). The top view and side view profile of the hexagonal arrangement of sub-wavelength cylinders shown in the inset. The height and diameter of the structure are: H = 710 nm, h = 0 nm, and D = 625 nm. (**b**) Selected transmission spectra for pitch, Λ = 0.8 μm (blue curve), 1.2 μm (red curve) and 1.6 μm (green curve) are shown.

The arrangement of individual scatterers into a periodic array of dielectric nanowire one-dimensional (1D) grating structures [38] or spherical [39], cylindrical [34,40] two-dimensional (2D) grating structures has been of interest to tailor the overall transmission or reflection spectra at the resonance wavelengths. Even though such transmit- or reflect-arrays are well known in the microwave frequency range [41], at optical frequencies such structures have been realized only recently with advancement in precision nano-fabrication techniques, such as electron-beam, optical interference, stepper-based lithography, nanoimprint and self-assembly techniques [42]. Furthermore, the recent research interest in the area of surface-relief sub-wavelength features to realize metasurfaces has also led to a resurgence of interest in guided-mode resonance structures and resonant metasurfaces for sensing and nonlinear optical applications. The consequence of scaling from an isolated sub-wavelength cylinder to hexagonal array of 2D cylinders is illustrated in the transmission contour map of Figure 4. For these simulations, rigorous coupled-wave analysis (RCWA) method was used to simulate the two-dimensional array using S4, electromagnetic solver package [43]. The pitch of the hexagonal array is increased from 0.8 to 2.4 micrometer, keeping the dimensions of the individual cylinders same as in Figure 3. For large separation between the cylinders, the individual disks do not interact with each other and the transmission spectra shows poor contrast and remains unchanged. The hexagonal array also acts as a higher order diffraction grating with significant energy being directed to non-zero diffraction orders. The transmission spectra used to plot the contour in Figure 4 do not separate the different diffraction orders and hence does not show this effect. With a reduction in separation distance, there is a more prominent interaction between the cylinders, which shows up as characteristic high contrast resonant features in the spectra. These high contrast resonant features are found to be in close vicinity to the peaks in the scattering spectra observed for the isolated cylinder case, as shown in Figure 3. This clearly shows that, though scattering from individual dielectric objects is the underlying reason for the frequency selective interaction with the electromagnetic field, the collective effect due to the array is important in determining the overall resonant spectral features and the associated field enhancement. Isolated sub-wavelength metallic and dielectric particles have found their own niche applications in in-vivo cell imaging [44], super-resolution microscopy [45] etc. For practical applications in photonic devices, and in particular in nonlinear optics, arrayed sub-wavelength objects and their characteristic spectral resonances are found to be more relevant when compared to isolated ones. However, it needs to be mentioned that the ability to achieve high-quality factor resonances in isolated structures utilizing bound-states in continuum mechanism, also called as super-cavity resonance is another promising direction of recent research in recent times [46].

### 2.2. Effect of Refractive Index Contrast on Resonant Interaction

**Figure 5:**
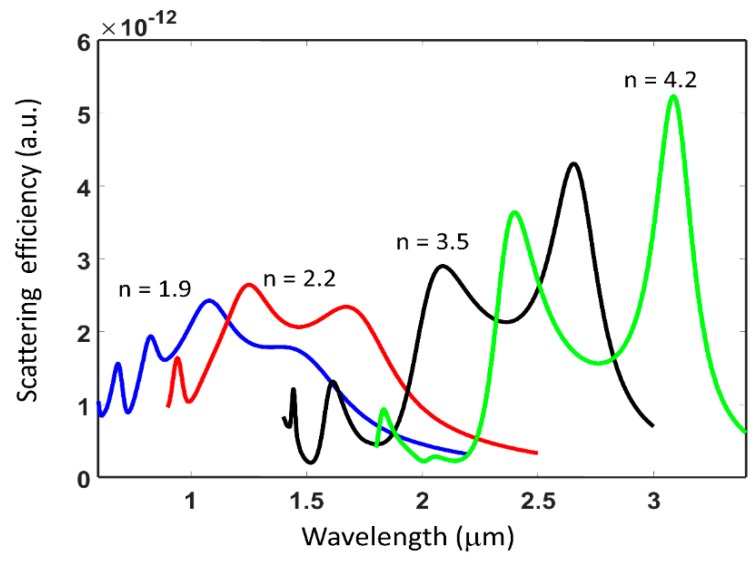
Scattering efficiency spectrum from isolated sub-wavelength dielectric sub-wavelength disk with dimensions same as in Figure 3 as a function of varying refractive index. Refractive index of 1.9 (blue curve), 2.2 (red curve), 3.5 (black curve) and n = 4.2 (green curve) are shown. The refractive index is assumed to be constant across the spectral range shown for each curve.

The refractive index of the sub-wavelength dielectric particle plays a critical role in determining the light scattering strength and hence its resonance characteristics. Figure 5 shows the scattering efficiency spectra for varying refractive indices for isolated dielectric sub-wavelength disk. A constant refractive index across the spectral range of interest and lossless dielectric medium are assumed in these simulations. The dimensions of the disk are same as in Figure 3. It is found that the contrast or sharpness of the resonances increases with increasing refractive index. This is associated with the higher quality factor of the resonance and stronger field concentration in the dielectric structure with higher refractive index. Furthermore, the scattering spectra is found to shift to longer wavelengths with increasing refractive index. This can be associated with the increased optical path length with increasing refractive index of the dielectric structure and its comparison relative to the wavelength range over which scattering is observed. 

A detailed comparison of the scattering spectra of plasmonic and dielectric sub-wavelength structures as a function of increasing dielectric constant is reported in ref. [47]. 

**Figure 6:**
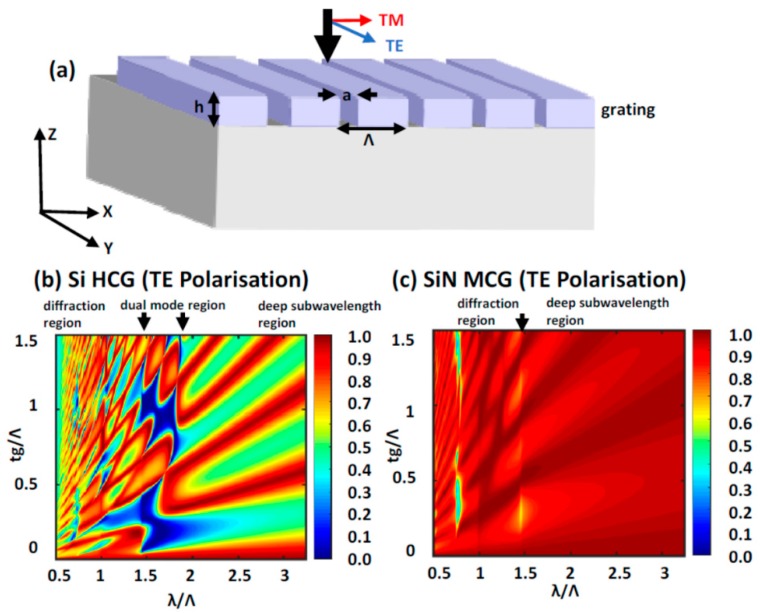
(**a**) Schematic of fully-etched dielectric one-dimensional grating structure. The simulated transmission spectra for (**b**) silicon high contrast grating and (**c**) silicon nitride medium contrast grating as a function of varying height for fixed incident TE polarization. The dimensions and wavelength are normalized by the pitch of the grating structure. (Figure is reproduced with permission from ref. [49]).

In the case of 1D sub-wavelength periodic grating structures, the role of the refractive index of the grating material on the resonant spectra are shown in Figure 6 [48,49]. A schematic view of the 1D grating structures simulated is shown in Figure 6a. The transmission spectra contour maps of fully-etched, high-index contrast grating structure of silicon and medium-index contrast grating structure of silicon nitride are compared for varying grating heights. The spectra are shown for incident transverse electric (TE) polarization, oriented parallel to the gratings. It is found that, for silicon high contrast grating structures, the transmission spectra show prominent resonance features [50], as evident from the checkerboard type patterns in Figure 6b. In contrast to this, the silicon nitride medium contrast gratings show poor contrast resonance features, as shown in Figure 6c with a reduced wavelength range over which the resonance features are observed. This is a direct consequence of the reduced spectral window between the zeroth order diffraction and first order diffraction into the glass substrate for the case of silicon nitride when compared to silicon. The wavelength range across which zeroth order diffraction occurs, also called as the dual-mode resonance is denoted by the black arrows in each of the contour maps is found to be reduced for the silicon nitride structures. Thus, high index contrast periodic structures are generally better suited for engineering optical resonances for nonlinear optical applications. However, they invariably end up being highly lossy in the visible and near infrared wavelength region due to enhanced absorption above their energy bandgap. The medium index contrast materials such as silicon nitride, titanium oxide etc. do offer certain benefits in terms of extended low-loss transmission window when compared to high-index contrast structures. For example, in the case of silicon nitride, the low-loss optical window extends from close to 300 nm to 5 μm. This is particularly beneficial to realize high quality resonances both at the fundamental excitation and nonlinear signal wavelengths. Thus, there is still interest in research into alternate structures based on guided-mode resonances, partially etched zero contrast grating structures etc. to obtain prominent resonances using medium index contrast material systems [48]. 

## 3. Physical Mechanisms behind Resonances in Arrayed Structures 

In this section, we discuss the underlying physical mechanisms that lead to resonances in the arrayed structures. Though the resonances in isolated and arrayed structures can be accurately modelled and designed based on scattering expansion or coupled-wave analysis methods as discussed in Section 2, these techniques do not give much physical insight into the working of the arrayed resonant structures and how incident light field interacts with the structures. Thus, it is instructive to describe the underlying working mechanism of the arrayed structures used for resonant nonlinear optical studies. Here, the resonance phenomenon studied are broadly classified as guided-mode resonances, EIT- like resonances and bound-states-in-continuum resonances. Though this list is not exhaustive [17,51], a majority of the resonant structures studied fall into these categories.

### 3.1. Guided-Mode Resonances

**Figure 7:**
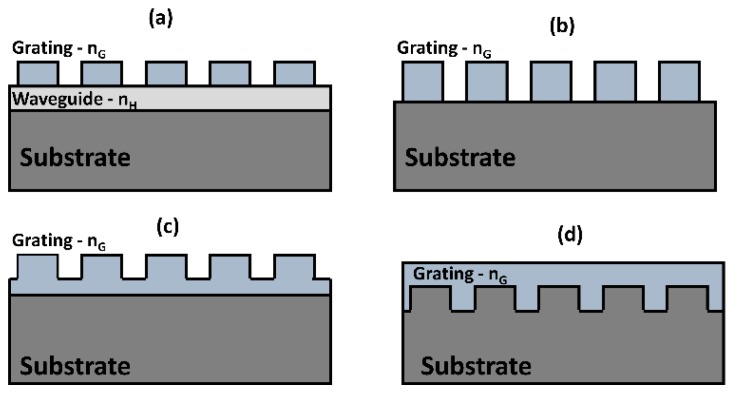
Cross-section view of various guided-mode resonance based grating structures with waveguide and grating layer refractive indices n_H_ and n_G_ respectively. (**a**) The grating and waveguide are made of different materials with the waveguide of higher index below the fully-etched grating structure. (**b**) Fully etched grating structures which can act as the effective waveguide. (**c**) Partially etched grating structures with the waveguide layer made of same material as the etched gratings. (**d**) Substrate grating structures is coated with a high-index waveguiding layer on top.

Guided-mode resonances arise in dielectric grating-waveguide coupled structures due to the evanescent diffraction orders from the grating coupling the incident electromagnetic wave into guided modes of the waveguiding layer. In other words, the optical resonances can also be described by the wavelength selective in- and out-coupling of electromagnetic wave into the waveguide through interaction with the grating structure. The waveguide can be either a separate high index layer located close to the grating structures or can be the grating structure itself which is fully or partially etched. Few examples of such structures which support guided-mode resonances in various waveguide-grating arrangement are shown in Figure 7. Here, we briefly outline some of the properties of periodic grating structures and demonstrated applications. Recent review articles provide a comprehensive overview of the recent advances in resonant waveguide grating structures [17,52].

**Figure 8:**
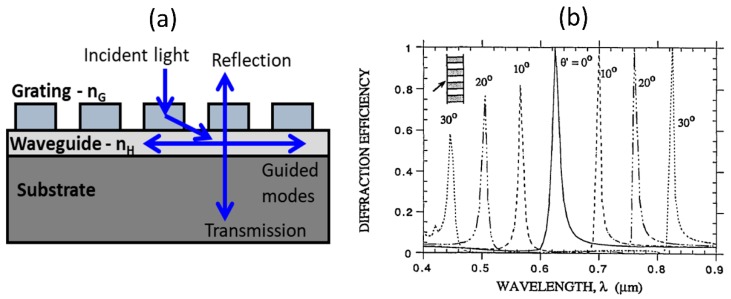
(**a**) A cross-section schematic of the guided-mode resonance structure showing the resonant coupling of incident light into the waveguide region through interaction with the grating structure. (**b**) Simulated filter response for the guided-mode resonance structure for parameters: pitch = 330 nm, height = 330 nm, dielectric constant difference (normalized), ∆ε/ε_avg_ = 0.05 and center wavelength of 547 nm and dependence of the filter response on the angle of incidence. (Figure b is reproduced with permission from ref. [38]).

Wood anomaly in diffraction gratings has been studied in the past to explain the occurrence of sharp spectral orders in the diffracted light [53]. In particular, resonance type anomalies are explained based on leaky guided-modes supported in the waveguide grating structures. A basic waveguide-grating model used to describe the diffraction effect from the periodically index modulated structure leading to coupling of waveguide modes is shown in Figure 8a [38,54]. This model is adapted from the seminal paper by R. Magnusson et.al. [38], which explained the working mechanism and applications of the guided-mode resonant structures. Under weak perturbation, the supported guided mode resonances can be understood based on the frequency-selective excitation of the modes of an effective waveguide formed by the periodically index modulated structure with the grating providing the required phase matching to couple the free-space incident light to the waveguide mode. Typical resonant filter characteristics obtained at normal incidence is shown in shown in Figure 8b [38]. Under weak index modulation, the filter reflection spectra are found to be strongly angle sensitive, as shown in Figure 8b. This offers a mechanism to angle-tune the filter characteristics. Such resonant response from waveguide-grating structures have been utilized as band-pass and band-stop filters working across different electromagnetic spectral regions. They have also been proposed to be used as intra-cavity narrow-band high-reflection mirrors, photorefractive tunable filters, and as electro-optic switches [38]. Guided-mode resonances have also found applications as bio-sensors in immune-assays in which local reaction between the functionalized antibody and the target antigen leads to a shift in the guided-mode resonance position [48,50,52,55]. Such assays have been proposed to be used in bright-field (colorimetric), fluorescence and also in wide-field imaging modes. In the context of nonlinear optics, the guided-mode resonance grating structure have been utilized as the nonlinear media in which the nonlinear optical interaction is enhanced due to the local electric-field built-up in the structure close to the resonance wavelengths. Some of these examples are discussed in Section 4 below.

**Figure 9:**
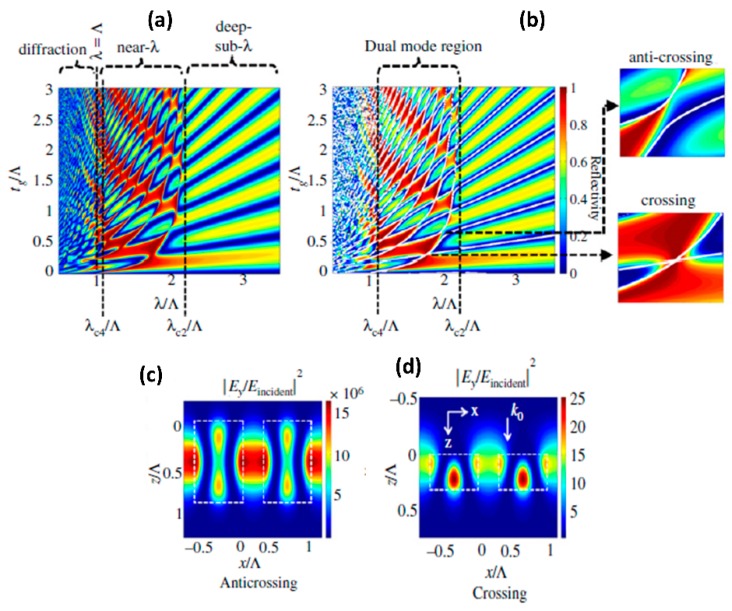
(**a**) Reflection spectra contour for free-standing silicon high contrast gratings with dimensions: n_grating_ = 3.48, duty cycle = 70% and thickness varied. The various regions of operation of the grating are also shown. (**b**) The reflection spectra overlapped with the solutions to the eigen-mode equations of the resonant modes (white curves). The overlap regions of the white curves result in anti-crossing and crossing type resonance. (**c**) Field intensity profile at anti-crossing resonance. (**d**) Field intensity profile at crossing resonance. (Figures are reproduced with permission from ref. [50]).

Fully etched high index contrast gratings, also termed as high-contrast gratings [50] are also used to create high quality factor resonances. A cross-section schematic view of such a structure is shown in Figure 7b above. The resonances in these structures can be understood either based on guided mode resonances with the grating coupling light into strongly-modulated effective waveguide created by the air/silicon periodic structure or based on optical modes supported in the periodic air-high index structure along the longitudinal propagation direction (i.e., along the optical axis) with energy exchanged with the interface due to reflection [56]. The resonant characteristics achieved in the high contrast grating structures have been classified as crossing and anti-crossing resonances based on the phase difference between the interacting longitudinal modes being odd and even multiple of π respectively [50,56]. The classification of the resonance spectra as crossing and anti-crossing features is shown in Figure 9. The anti-crossing resonances exhibit highly asymmetric Fano-like line shape with strong-coupling between the interacting modes. This results in strong field enhancement within the structure as shown in Figure 9c with high quality factor. In contrast, the crossing resonances exhibit more symmetric spectral shape, however with lower quality factor and reduced field enhancement, as shown in Figure 9d. The high contrast gratings have been used to build both narrow-band and broad-band reflectors with reflectivity greater than 95% for use as end-facet mirrors in vertical cavity surface-emitting lasers (VCSELs) [50]. They have also been used as bio-sensors based on refractive index changes altering the resonance characteristics [55]. The strong field-concentration has also been utilized for Raman scattering enhancement with suitable metal nanostructures patterned in regions of strong dielectric field concentration [57]. In the context of nonlinear optics, the strong nonlinear optical properties of the high index materials, such as Aluminum Gallium Arsenide and Silicon are leveraged in enhancing second-harmonic generation and four-wave mixing due to strong field enhancement near anti-crossing resonances [58,59]. This is further discussed in Section 4 below.

**Figure 10:**
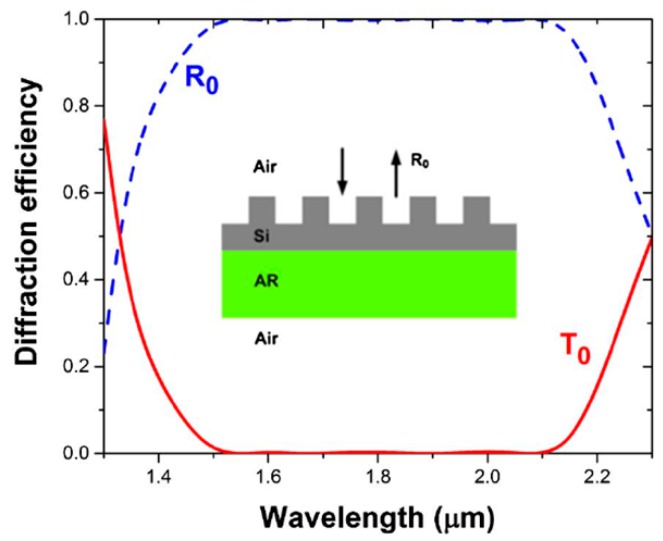
The simulated zeroth order reflection (R_0_) and transmission (T_0_) spectra for silicon zero-contrast gratings. The inset shows the cross-section of the zero-contrast gratings with the dimensions optimized using particle swarm combined with inverse-design algorithm. The optimized dimensions of the structure obtained are: etched grating height of 490 nm, unetched slab thickness of 255 nm, pitch of 827 nm, and fill factor of 0.643. (Figure reproduced with permission from ref. [60]).

In comparison to fully-etched, high contrast grating structures, partially etched structures, also termed as zero-contrast gratings [60,61], offer an additional degree of freedom for designing the grating characteristics based on the chosen etch depth. Such structures can achieve robust spectral characteristics comparable to, if not better than that of high contrast grating structures. As an example, the realization of broadband reflective filter using zero-contrast gratings is shown in Figure 10 [60]. The structures shown here have been optimized using an inverse design approach in which particle swarm search algorithm is utilized to quickly search a wide design space with the performance optimized based on achieving a desired value of figure-of-merit. In this example, the figure-of-merit is chosen as follows [60]:

(4) $$Figure\;of\;merit={\left\{\frac{1}{M}\overset{M}{\underset{i=1}{\sum }}\left[R_{desired}\left(\lambda_{i}\right)-R_{design}\left(\lambda_{i}\right)\right]\right\}}^{1/2}$$

The figure of merit compares the root-mean square error between the desired and designed reflection spectra with the goal of minimizing the difference between the two through optimized design. With multiple iterations this process helps optimize the device design to achieve the designed spectral response as close to the desired one as possible. A comparison of broadband reflector performance of zero-contrast gratings with high contrast gratings can also be found in ref. [60]. Such inverse design approaches are most promising to search a wide design space and at the same time achieve close to the ideal response for the resonant structure. Another emerging direction is the use of neural networks for optical metasurface design optimization [62,63] to achieve optimal specifications with often non-intuitive, but effective meta-atom shapes which can be fabricated with present-day advanced nanolithography tools. 

The angle sensitivity of the guided-mode resonance filter is another useful characteristic for filter response tuning or for wide field of view application. This is also found to be a strong function of the effective index of the waveguide-grating structure, with higher index resulting in stronger field confinement in the unit-cells and hence less angular sensitivity [50]. In this context, high contrast gratings with highly confined field profiles within the etched structures are found to be more angle insensitive than the partially etched zero-contrast gratings [50,64], which support diffused field-profiles extending across the unetched high-index slab region. Furthermore, conical mounting of partially etched gratings is also found to result in reduced angle sensitivity when compared to conventional mounting [65]. One-dimensional grating structures discussed above are inherently polarization selective and polarization independence is achieved by using two-dimensional symmetric meta-atoms, such as square or hexagonal arrangement of circular features. Such polarization independent structures are particularly useful for realizing optical filters for unpolarized light [66] or for realizing resonantly enhanced fluorescence sensors [48]. 

### 3.2. Electromagnetically-Induced Transparency Analogue Resonances

**Figure 11:**
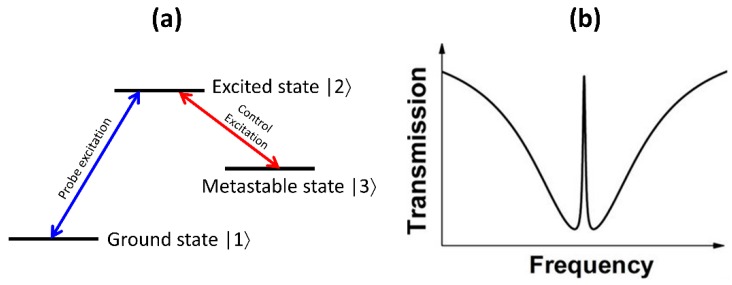
(**a**) The representative energy diagram for electromagnetically-induced transparency (EIT). (**b**) Schematic of the expected transmission spectrum for the EIT effect.

Optical resonances in photonic structures can be created due to interference between coupled resonances of varying quality factor. For example, a high-quality factor and low-quality factor resonance can couple together to result in the observation of analogues of electromagnetically-induced transparency (EIT) in photonic structures [18]. The electromagnetically-induced transparency effect can be understood based on quantum interference between two different path-ways to excite an upper energy level in a three-level system, as shown in Figure 11a. This system consists of a lower level, an upper level and a meta-stable state, as denoted in the figure. A probe field can control the transition from the lower to upper state, while a control field can control the transition from the excited state to metastable state. The direct transitions from the lower state to metastable state are forbidden. In this context, the transitions induced by the probe field can interfere with the indirect transitions from lower to upper state through interaction with the metastable state, in the presence of a strong control field. This can result in destructive interference of the probe field and results in the observation of peaks at the probe field frequency in an otherwise expected dip in transmission spectrum, as shown in Figure 11b. This phenomenon is hence termed as electromagnetically induced transparency. In the context of photonic systems, the low-quality factor and high-quality factor resonant modes are equivalent to the direct and indirect lower level to upper level transition respectively, as discussed above. 

**Figure 12:**
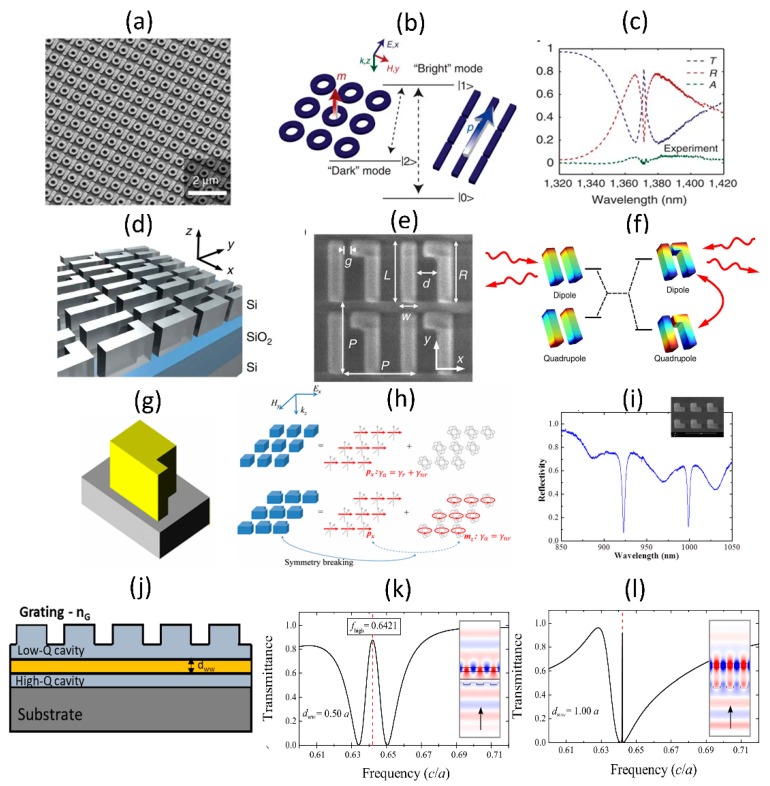
Different implementations of EIT-like resonance. (**a**) SEM image of bars-ring array. (**b**) Schematic of the coupling between the bar excited by the incident light and coupling to the ring structure creating magnetic dipolar type mode. (**c**) Experimentally measured transmission spectrum for the bar-split ring array showing EIT-like resonance. (**d**) Schematic of the achiral bar-dimer structures. (**e**) SEM image of asymmetric dimer achiral structures. (**f**) Coupling between the dipole and quadrapolar modes in the asymmetric dimer structures. (**g**) Perspective view of the asymmetric nanocube unit-cell. (**h**) Schematic showing the coupling of electric and magnetic dipole modes in asymmetric nanocubes. (**i**) Measured reflection spectrum for the asymmetric nanocube array. (**j**) Cross-sectional view of the structure showing upper layer grating-waveguide structure coupled to lower waveguide structure. (**k**,**l**) Simulated EIT-like resonance spectra and associated field profiles from GMR structures. (Figures a–c are reproduced with permission from ref. [68], d–f are reproduced with permission from ref. [70], g–i are reproduced with permission from ref. [72], k,l are reproduced with permission from ref. [74]).

Photonic structures in the form of coupled Fabry-Perot cavities, coupled ring resonators, coupled photonic crystals etc. have been explored to be studied as EIT analogues [18]. In the context of arrayed metasurfaces and guided-mode resonant structures, there is also interest in coupling bright-mode and dark-mode resonances to observe EIT effects. Few examples of such implementations, supporting simulations and experimental studies are shown in Figure 12. Figure 12a–c shows an array of bar-ring structures in which the incident linearly polarized light preferentially couples to the bar and indirectly couples into the ring through the bar excited mode [67,68]. This results in excitation of dark modes in the ring, as shown in Figure 12b. The measured transmission spectra show peak in the middle of a broad transmission dip with strong field localization in the ring structures with maximum quality factor of the EIT resonance of ~300 times. Asymmetry in the array elements also results in coupling between bright and dark modes. Figure 12d–f shows one such example of rectangular bar dimer array with slight asymmetry added to one of the bars [69,70]. In this case, the coupling between the bright dipolar mode with the dark quadrapolar modes results in peaks in the transmission spectra (shown in Figure 12f) in the mid infrared wavelength region. Such structures have been used for enhanced infrared sensing [71]. Asymmetry can be introduced to the shape of a single monomeric unit as well to achieve bright-to-dark mode coupling. This is shown in Figure 12g–i, for the case of a nanocube with symmetry breaking protrusion [72]. As shown in Figure 12h, this protrusion results in the coupling of the electric dipole excitation, denoted as *p_x_* to the magnetic dipole mode, denoted as *m_z_*. This coupling results in characteristic dips in the measured reflection spectra, as shown in Figure 12i. Such structures have been utilized for nonlinear optical process enhancement, as discussed in Section 4. In the context of guided mode resonances, there is interest in coupling a low- and high- quality factor resonant waveguide structure. Some examples of such structures are shown in Figure 12j–l. Some of the early simulation studies of these structures consisted of a top grating-waveguide layer coupled to another bottom waveguide layer, as shown in the inset of Figure 12j. In this case, the direct coupling of the incident light to the bottom waveguide and indirect coupling through interaction with the top-layer guided-mode resonant structure can occur. This results in a sharp transmission peak, which is different from the transmission spectra of an equivalent refractive index homogenous medium. Some of the early work [73] was not even called an EIT analogue at that time. The EIT analogue ideas have been extended recently to one-dimensional and two-dimensional structures [74,75]. The spectral width of the EIT resonance and hence its overall quality factor can be engineered by choosing the separation between the top-layer grating-waveguide structure and the bottom waveguide layer. An example of such an engineered resonance shape is shown in Figure 12k,l. Such resonance shapes can potentially be used as narrow band-pass filters when compared to the more complex multi-layer dichroic filters.

### 3.3. Bound-States in Continuum Resonances

**Figure 13:**
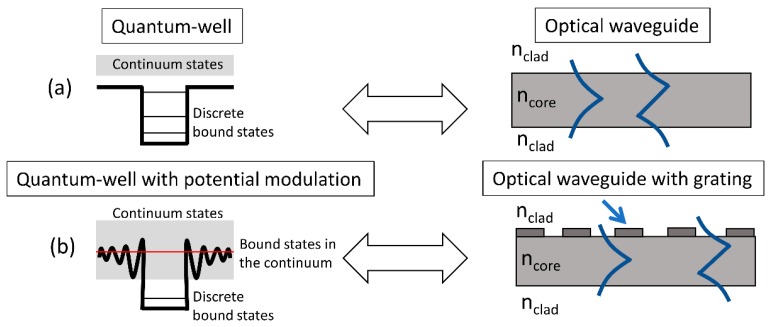
(**a**) Schematic of a quantum well with sharp potential edges and the corresponding photonic analogue showing an optical waveguide with confined modes, (**b**) Schematic of quantum well with modulated edges and the corresponding photonic analogue showing grating-modulated waveguide. (Figure adapted from ref. [76]).

Bound-states in continuum (BIC) represent another interesting class of high-quality factor resonance. BIC are bound states in an otherwise continuum of states, considered as a trapped state with embedded eigenvalue. Figure 13 shows a schematic representation of BIC states in quantum-well systems and comparison with analogous waveguide grating systems (figure adapted from ref. [76]). Figure 13a compares a conventional quantum well with a slab-waveguide. It is found that in a conventional quantum well, the allowed states within the quantum well are bounded by the steep potential walls of the quantum well. This results in discrete, bounded states within and a continuum of states outside. This can be compared with a straight, unperturbed slab waveguide with optical mode confinement achieved by the core-cladding refractive index profile. This results in discrete, bound modes within the waveguide and a continuum of radiation modes outside. When the sharp edges of the quantum well potential profile are replaced by a modulated potential profile, it is found that there can exist bound states even within the continuum, as shown in Figure 13b. In similar lines, the introduction of a periodic modulation to the waveguide using a grating structure results in the creation of bound-states within the continuum of radiation states. These states end up being forbidden from external excitation/ coupling due to symmetry considerations [77]. This lack of in- and out-coupling or non-radiating characteristic can result in ideal infinitely high-quality factor for these bound states within the continuum resonances. However, in practice one would consider quasi-BIC states which tend towards the ideal situation and can result in large, but finite quality factors with external excitation mechanism [78,79]. 

**Figure 14:**
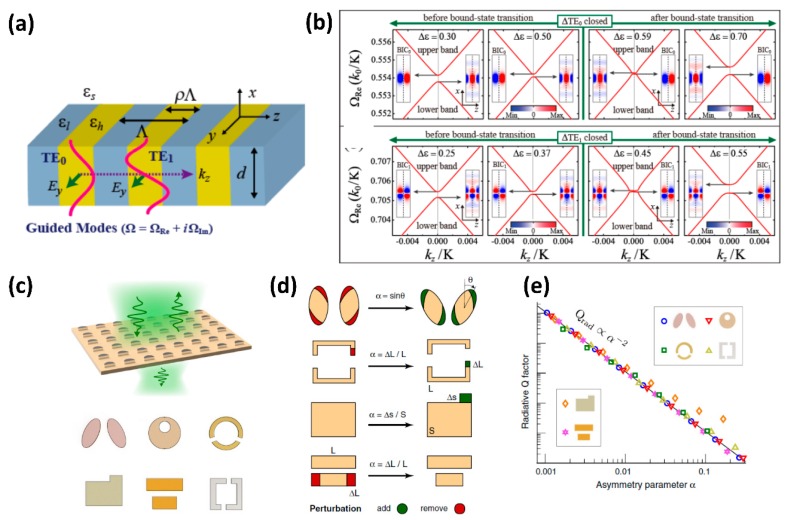
(**a**) Schematic of the periodic dielectric constant modulated grating structure with typical TE0 and TE1 modes supported by such structure. (**b**) Photonic band-structure calculation corresponding to the TE0 and TE1 modes showing the GMR and BIC states at either band-edge and their corresponding mode profiles. The GMR and BIC states are found to flip by changing the dielectric constant difference between the grating materials. (**c**) Examples of asymmetric resonant metasurfaces which support quasi-BIC resonances. (**d**) Modeling the asymmetricity using an asymmetry parameter, α. (**e**) Variation of quality factor of the quasi-BIC resonance with change in asymmetry parameter. (Figures a–b are reproduced with permission from ref. [81], c–e are reproduced with permission from ref. [83]).

The BIC states in the context of periodic grating structures can be broadly classified as symmetry protected BIC and accident BIC [76]. Symmetry protected BIC occurs at the high symmetry, zero wave-vector points (Γ-point in the case of photonic band structures) with direct excitation forbidden for normal incidence by the BIC resonant field symmetry. An example of such symmetry protected states in a simulated periodic dielectric constant modulated structure is shown in Figure 14a [80,81]. The schematic shows the periodic modulated structure with dielectric constant difference of Δ*ε* = *ε_H_*−*ε_L_*. The structure supports multiple resonant modes (TE_0_ and TE_1_ modes) as shown in the figure. The bandstructure for the TE0 and TE1 resonances in the vicinity of k_z_ = 0 point shows two different types of resonances, one which is the leaky GMR and the other is the non-leaky BIC resonance [82], as shown in Figure 14b. The mode profile of the GMR and BIC resonances show odd and even order symmetry respectively and this inherently determines the ability to excite or couple into these modes through normal incidence plane wave excitation. The odd-symmetry profile can be excited with a normal incident wave, while the even-symmetry profile is forbidden from excitation. Furthermore, the GMR and BIC resonances are found to flip with change in Δ*ε* [80]. The same band dynamics are observed for both TE_0_ and TE_1_ resonant modes. The symmetry protected BIC resonances strictly remain protected only at normal incidence. With off-axis illumination, the symmetry can be broken resulting in quasi-BIC resonances with finite quality factor. BIC resonances can also be observed for non-zero k_z_, which are called accidental BIC resonances [76]. Quasi-BIC resonances can also be excited at normal incidence by the introduction of asymmetry in the periodic structures [83]. Figure 14c shows schematic of such asymmetric structures. The resonant metasurfaces can be modelled by the amount of asymmetry introduced into the structure, denoted by α parameter [83]. Figure 14d shows that the asymmetry parameter can represent angular tilt, addition/ removal of material in split-ring, rectangular and bar-dimer structures in normalized units [83]. The asymmetric resonant metasurfaces discussed in Figure 12g–i which exhibit EIT-like coupling between the electric and magnetic dipolar modes can also be considered as an asymmetric structure in which quasi-BIC modes are observed. The quality factor of the BIC resonance is found to be directly related to the asymmetric parameter, with the quality factor scaling as α^−2^ [83]. In addition to resonant metasurfaces, BIC resonances are also predicted for isolated sub-wavelength particles in the form of narrow spectral features in the scattering spectra. These are termed as super-cavity modes [46]. Such high-quality factor BIC resonances in periodic grating structures, asymmetry metasurfaces and even isolated objects are finding innovative applications in BIC metasurface lasers [84,85], sensing [86], and nonlinear optics [87]. Few of the nonlinear optics applications are discussed below in Section 4.

## 4. Nonlinear Optical Studies of Resonant Dielectric Grating Structures

In this section, the various nonlinear optical processes studied in the context of guided-mode resonance structures and resonant metasurfaces are discussed. These are broadly classified based on the complexity involved in terms of the nonlinear optical processes studied or the structure being considered for this study. First, the basic nonlinear harmonic generation processes such as second and third harmonic generation are considered, following which wave-mixing processes such as four-wave mixing and sum-frequency generation are considered. This is followed by ultra-fast optical switching, photon acceleration effect, and higher harmonic generation processes. Lastly, nonlinear optical studies in hybrid metasurfaces are discussed. The discussion is aimed at outlining the salient features of the respective nonlinear processes and specific structures studied. There are few previous review articles written in the areas of nonlinear metasurfaces in plasmonic [9,11], dielectric metasurface [14] and guided-mode resonance [17] platforms. There is also interest in utilizing the metasurface to shape the wavefront of the generated nonlinear signal for beam steering or focusing applications [88,89,90]. These efforts are not discussed here to keep the focus solely on the resonant enhancement of nonlinear optical processes. 

### 4.1. Second- and Third-Harmonic Generation

Second and third-order nonlinear optical processes are considered as the basic nonlinear optical processes studied in optical media under the influence of a strong incident electric field. The induced polarization or the response of the medium to the incident electric field can be expanded in a perturbative approach into various nonlinear optical processes as follows [1]:

(5) $$\vec{P}\left(\omega_{out}\right)=\varepsilon_{o}\left(\chi^{\left(1\right)}.\vec{E}\left(\omega \right)+\chi^{\left(2\right)}:\vec{E}\left(\omega \right)\vec{E}\left(\omega \right)+\chi^{\left(3\right)}\vdots \vec{E}\left(\omega \right)\vec{E}\left(\omega \right)\vec{E}\left(\omega \right)+\cdots \right)$$

**Figure 15:**
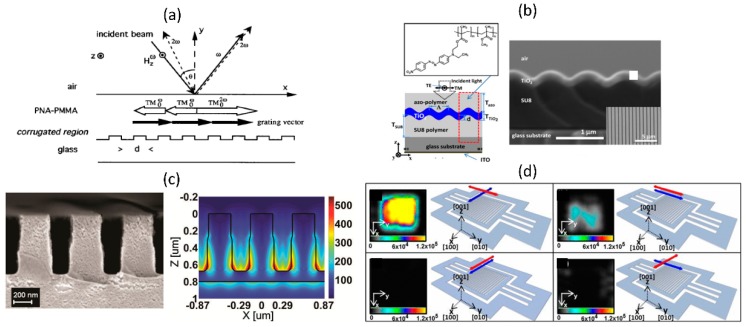
Various guided-mode resonance structures studied for nonlinear optical enhancement studies. (**a**) Schematic of glass-grating with PMMA layer used for phase-matched second-harmonic generation studies. (**b**) Schematic and scanning electron microscopy image of the Azo-polymer coated titanium oxide gratings used for second-harmonic generation enhancement. (**c**) Silicon nitride grating structures and simulated field profiles used for UV-third-harmonic generation. (**d**) AlGaAs high-contrast grating structures and second harmonic generation microscopy studies for different incident/ detection polarizations. (Figure a is reproduced with permission from ref. [92], b is reproduced with permission from ref. [93], c is reproduced with permission from ref. [95] and d is reproduced with permission from ref. [96]).

**Figure 16:**
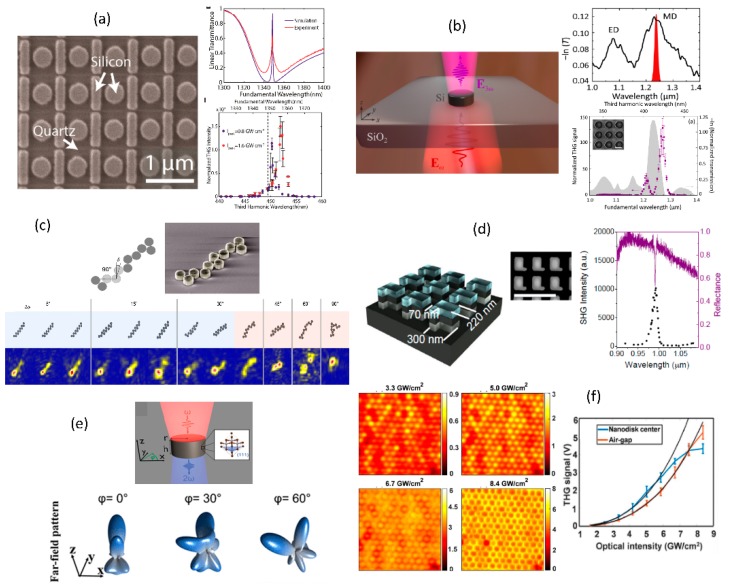
Various implementations of resonant metasurface for second- and third-harmonic generation studies. (**a**) Fano-resonant silicon bar-nanodisk structures for third-harmonic generation enhancement. (**b**) Silicon nanodisk array for third harmonic generation enhancement relying on magnetic dipolar modes. (**c**) Disorder robust third-harmonic generation from silicon nanodisks which are shown to be topologically protected from disorder in arrangement of the structures. (**d**) Gallium Arsenide asymmetry resonant metasurface for second-harmonic generation enhancement. (**e**) Dependence of the resonant second-harmonic far-field signal on [1 1 1] oriented Gallium Arsenide metasurface. (**f**) Spatial mapping of intensity dependent saturation of third-harmonic signal from silicon nanodisk array. (Figure a is reproduced with permission from ref. [97], b is reproduced with permission from ref. [34], c is reproduced with permission from ref. [100], d is reproduced with permission from ref. [102], e is reproduced with permission from ref. [103] and f is reproduced with permission from ref. [104]).

The nonlinear interactions which depend quadratically and cubically with the incident electric field gives rise to second- and third-order nonlinear processes respectively. The strength of the nonlinear optical processes can be enhanced significantly by the enhancement of the incident electric field inside the resonant metasurface. The nonlinear process typically scales as the (Q/V)^n^ where, Q is the quality factor of the resonance under consideration and V is the cavity volume and n is the order of the nonlinearity [91]. With reduced cavity volumes in sub-wavelength metasurfaces and the ability to achieve moderately high-quality factors (few 100 s to 1000 s), the resonant nonlinear optical process can be enhanced by 10^3^ to 10^5^ times. This field enhancement can counteract the effect of reduced interaction length in sub-wavelength thick metasurface, which is potentially promising for realizing high efficiency nonlinear photonic devices. Second order nonlinear optical processes are observed in materials which lack inversion symmetry and in material interfaces, while third-order nonlinear optical processes are observed in all optical media [1]. This leads to the careful selection of the nonlinear media to build resonant metasurface platforms for study various nonlinear optical processes. In general, the second- and third- harmonic generation processes satisfy the frequency relationships, $\omega_{out}=\omega +\omega \;$ and $\omega_{out}=\omega +\omega +\omega$ respectively. The need for momentum or wave-vector matching is relaxed in sub-wavelength metasurface platforms in most implementations due to the reduced length resulting in negligible phase mismatch. Here, we broadly divide the second and third-harmonic generation studies in periodic dielectric structures into guided-mode resonance type and resonant metasurface type platforms. Few examples under each of these categories are listed in Figure 15 and Figure 16, respectively.

Some of the early sub-wavelength periodic structures studied for nonlinear optical applications are the guided-mode resonance structures leveraging the resonances offered by the dielectric grating structures to enhance nonlinear effects from nonlinear polymer overlayers. Figure 15a,b show two such implementations using PMMA [92] and Azo-polymers [93] as the nonlinear media on top of glass and titanium oxide gratings respectively. In Figure 15a, careful attention is paid to the phase matching of the second-harmonic generation process between the counterpropagating fundamental and second-harmonic slab modes in the presence of the grating structure [92]. Experimentally measured second-harmonic signal shows enhancement corresponding to the phase matched condition when compared to the non-phase matched case. The use of higher refractive index gratings, such as periodically patterned titanium oxide layer is found to enhance the local electric field in comparison to the glass gratings and this is found to enhance second-harmonic by ~3500 times from a Azo-polymer overlayer when compared to a reference sample without the guided-mode resonance structures [93]. There has also been interest in studying nonlinear optical processes from the guided-mode resonance grating structures itself. In this context, silicon nitride gratings have been used for second- and third-harmonic generation studies [94,95]. Even though the nonlinearities in silicon nitride is weak and the index contrast with the substrate is small, the broad optical transparency window from the visible to mid infrared, makes it attractive for realizing high quality resonant structures. An example for the use of silicon nitride sub-wavelength grating structures for third-harmonic generation in the ultraviolet spectral region is shown in Figure 15c [95]. Aluminum Gallium Arsenide (AlGaAs) high-contrast grating structures with characteristic optical resonances have also been explored for second-harmonic generation studies. Schematic images of such free-standing AlGaAs high contrast grating structure and the corresponding second-harmonic microscopy images obtained for different orientations of the fundamental and second-harmonic polarization are shown in Figure 15d [96]. 

In the context of resonant metasurfaces for second- and third- harmonic generation studies, sub-wavelength spaced arrays of high-index semiconductors such as silicon, germanium and gallium arsenide have been studied. Fano-resonances from silicon bar-nanodisk structures, similar to the bar-ring structures shown in Figure 12a have been utilized to enhance third-harmonic generation [97]. The scanning electron microscopy image and the measured linear and third-harmonic spectra are shown in Figure 16a. Maximum third-harmonic signal enhancement of ~10^5^ has been reported in this work with an overall conversion efficiency of 10^−4^. Silicon nanodisks in ordered two-dimensional arrangement have been used to leverage magnetic dipolar resonances from the unit cell elements to enhance third-harmonic generation [34]. Figure 16b shows one such arrangement of nanodisks with the corresponding linear and nonlinear spectral measurement results. Maximum enhancement of close to two orders of magnitude with conversion efficiencies of ~10^−7^ has been reported in this work. Similar third-harmonic enhancement studies have been extended to dimer and more complex oligomeric unit cells to study the collect interaction of the individual elements in the unit cells [98,99]. There has also been interest in understanding the effect of disorder in the particle arrangement [100,101]. Figure 16c shows the arrangement of the nanodisks with controlled disorder introduced during fabrication. In this work, it has been found that the third-harmonic signal and its spatial localization are robust against disorder added to the nanodisk arrangement, making it topologically protected. Gallium Arsenide metasurfaces have been used for second-harmonic generation enhancement [102]. Asymmetric metasurfaces with high quality factor utilized for one such work with the corresponding linear and nonlinear optical spectra are shown in Figure 16d. It is found that the common [1 0 0] oriented Gallium Arsenide results in negligible second-harmonic emission along the optical axis due to the dominant longitudinally polarized nonlinear polarization, thus resulting in poor collection efficiency. One way to alter the far-field emission profile is to change the Gallium Arsenide orientation [103]. Figure 16e shows one such work on nanodisk arrays of [1 1 1] Gallium Arsenide metasurfaces. It is found from the far-field angular distribution that [1 1 1] metasurface does result in strong second-harmonic emission parallel to the optical axis when compared to [1 0 0] metasurface. 

Spatially resolved nonlinear optical studies or nonlinear optical microscopy is also a useful tool to understand the spatial dependence of the nonlinear signal across different regions in the metasurface sample to understand the signal uniformity and can combined with spectral and intensity studies to understand spatial, spectral resonance and intensity saturation behavior of the nonlinear medium. In this context, Figure 16f shows the intensity dependence of the third-harmonic generation microscopy images across a silicon nanodisk array sample at its fundamental resonance wavelength [104]. It is found that the contrast in the third-harmonic microscopy images reverses with increasing intensity. This is attributed to the spatial position dependent onset of saturation of the third-harmonic signal as shown in the intensity dependent third-harmonic plot in Figure 16f. 

### 4.2. Wave Mixing Processes

**Figure 17:**
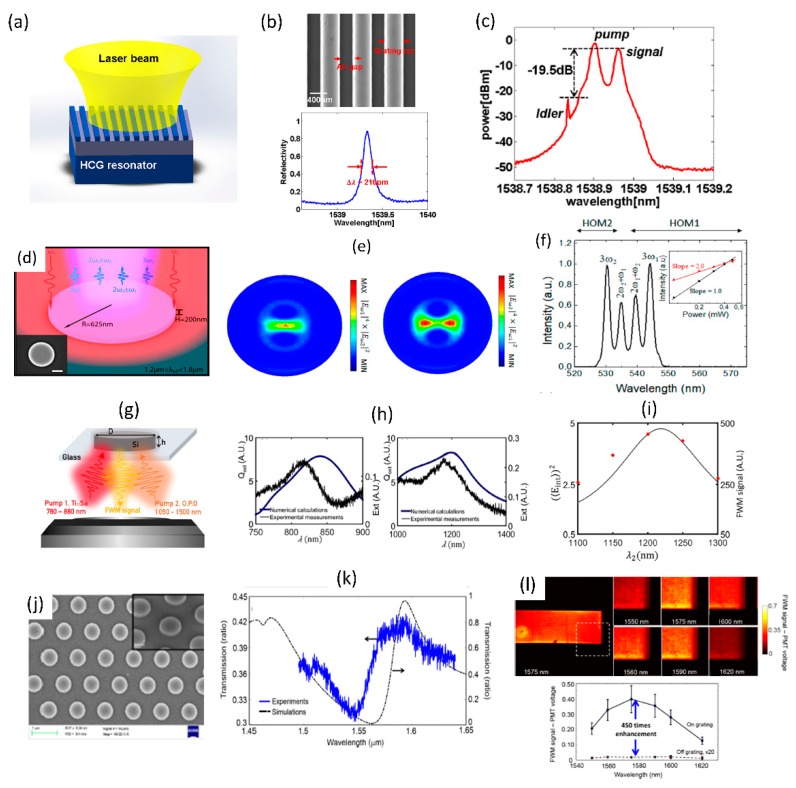
Various implementations of resonant FWM processes. (**a**) Schematic of high-contrast grating resonance, (**b**) Electron microscopy images and measured reflectivity spectrum of the resonance. (**c**) Measured FWM spectrum for the high-contrast grating structure. (**d**) Schematic of the high-aspect ratio Ge nanodisks for SFG studies. (**e**) Mode profiles of the nonlinear polarization for the two different SFG processes. (**f**) Measured SFG and THG spectra for the high-aspect ratio Ge nanodisks. (**g**) Schematic of Si nanodisk structures used for doubly-resonant FWM studies. (**h**) Comparison between measured and simulated scattering spectra for the two resonant modes under consideration. (**i**) Comparison of the measured FWM signal and simulated pump intensity enhancement as a function of wavelength. (**j**) Electron microscopy image of the partially etched zero-contrast grating structures used for FWM studies. (**k**) Simulated and measured transmission spectra for the zero-contrast grating structures. (**l**) FWM microscopy images for varying signal wavelength. The enhancement spectrum is also shown. (Figure a–c are reproduced with permission from ref. [105], d–f are reproduced with permission from ref. [107], g–i are reproduced with permission from ref. [108] and j–l are reproduced with permission from ref. [109]).

The wave-mixing processes can be considered as a general case of the above described harmonic generation processes. The processes of interest here are the four-wave mixing (FWM) and sum-frequency generation (SFG). In the case of FWM process, the nonlinear signal frequency is related to the incident light as follows: *ω*_4_ = *ω*_1_ + *ω*_2_ − *ω*_3_ [1]. Two pump frequencies (ω_1_ and ω_2_) being unique or identical are termed as non-degenerate and degenerate FWM processes respectively. The second-order SFG process satisfies the frequency relation: ω_3_ = ω_1_ + ω_2_, while degenerate third-order SFG process satisfies frequency relation of the form: *ω*_3_ = 2*ω*_1_ + *ω*_2_ or *ω*_3_ = *ω*_1_ + 2*ω*_2_ [1]. Figure 17a–c shows FWM enhancement observed for closely spaced pump-signal wavelengths in the telecom range for silicon-on-insulator based fully etched high-contrast gratings [105]. The sub-wavelength dimension high-contrast grating structures are found to support resonances with intensity enhancement of more than 8000 times and experimentally measured quality factor of ~7300. The signal and pump photons in close vicinity to this resonance results in FWM with the generation of idler with conversion efficiency of −19.5 dB as shown in Figure 17c. The use of high aspect ratio germanium (Ge) nanodisks to observe anapolar resonances [106] and the enhancement of third-order sum-frequency generation processes is shown in Figure 17d–f [107]. The higher order anapolar mode profiles are chosen with good spatial overlap to ensure enhancement of the SFG process by about two-orders of magnitude, as shown in the SFG spectrum in Figure 17f. Silicon (Si) nanodisks that support magnetic and electric-dipole resonances have also been utilized for doubly-resonant enhancement of FWM process as shown in Figure 17g–i [108]. The individual resonance spectra and the corresponding resonance for the FWM are also shown, with approximately two-orders of magnitude enhancement. Doubly-resonant structures are promising to increase the FWM efficiency using both pump and signal resonances. However, the best enhancement can be obtained only when good overlap is ensured between the interacting resonant mode profiles. Detailed spatially-resolved imaging of four-wave mixing process in singly resonant partially etched zero-contrast grating structures is shown in Figure 17j–l [109]. The structures are designed to support resonance at the signal wavelength in the 1550 to 1600 nm wavelength range. Four-wave mixing images acquired across an area of 300 × 300 microns show clear dependence of the FWM image contrast on the incident signal wavelength. A maximum FWM enhancement of 450 times has been experimentally obtained [109]. 

### 4.3. Optical Switching

**Figure 18:**
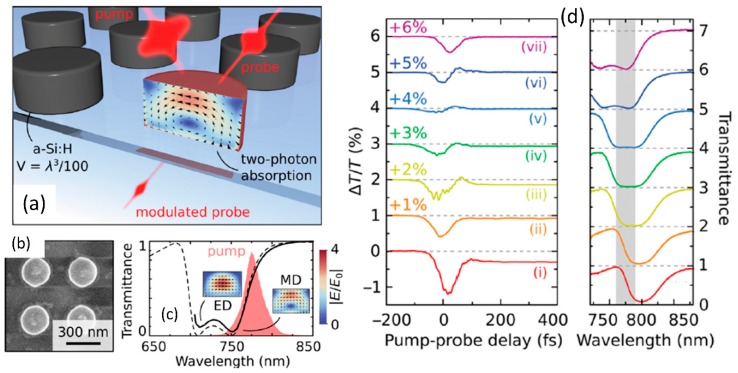
(**a**) Schematic of the nanodisk array used for ultrafast-optical switching studies. (**b**) Electron microscopy image and (**c**) transmission spectra for the nanodisk array with the corresponding dipolar modes marked. (**d**) Experimental results of pump-probe studied showing fast recovery or switching of probe in the presence of a resonant pump. Various pump laser wavelengths relative to the resonance are shown in the right plot. (Figures are reproduced with permission from ref. [110]).

The ultrafast Kerr nonlinearity and multi-photon absorption processes due to the nonlinear interaction of valence electronics in the dielectric medium with incident light can be used to perform fast optical switching at hundreds of femtosecond time scales. Such ultrafast switches have been demonstrated previously in guided-wave systems such as optical fibers and integrated waveguides utilizing self-phase and cross-phase modulation effects [2]. The optical resonances in dielectric resonant metasurfaces can be used to enhance the optical switching process through the enhancement of the nonlinear optical effect [110]. In the context of semiconductor metasurfaces, the presence of parasitic processes such as thermo-optic effects results in additional phase shift, albeit at much longer time scales. Figure 18a,b shows a schematic and scanning electron microscopy image of a silicon nanodisk array used for optical switching studies. The corresponding transmission spectra from the nanodisk array and the associated dipolar resonant modes are shown in Figure 18c. The optical switching process can be studied in a pump-probe configuration with a strong pump on-resonance leading to enhanced electric-field inside the nanodisks which results in a fast transmission dip at the probe wavelength due to enhanced multi-photon absorption. The fastest switching characteristics is obtained with a temporal response time of 65 fs with a slower extended recovery due to thermal and free-carrier recombination effects. Such resonant metasurface with ultrafast all-optical switching capability can find possible applications as fiber-connectorized photonic structures for high speed data communication and pulse-shaping [2].

### 4.4. Photon Acceleration

**Figure 19:**
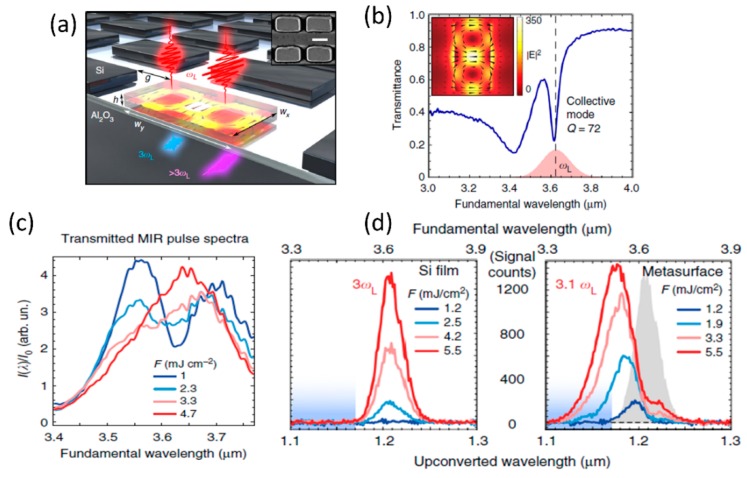
(**a**) Schematic of the silicon rectangular metasurface used for photon acceleration studies. (**b**) Measured transmission spectrum and field profile at resonance (inset). (**c**) The fundamental laser spectra transmitted through the metasurface for varying laser fluence. (**d**) Comparison of the third-harmonic signal generated for varying fundamental laser fluence for the un-patterned silicon film and silicon metasurface. (Figures are reproduced with permission from ref. [112]).

Time-dependent optical properties in a resonant medium can lead to noticeable spectral shift of propagating laser pulse and can manifest as wavelength shifts of corresponding nonlinear optical signals as well. Such spectral shifts of light in the presence of time-varying optical processes is termed as photon acceleration and has been studied previous in plasma media [111]. Similar effect have recently been observed in silicon based resonant metasurface due to shift in the resonant wavelength with increasing incident light fluence due to time-dependent free-carrier accumulation [112]. Schematic of the silicon-rectangular structures used to study photon acceleration is shown in Figure 19a. The corresponding transmission spectrum showing the optical resonance in the mid infrared wavelength range close to 3.6 μm and the corresponding field profile are shown in Figure 19b. With increasing incident laser fluence, the transmitted laser spectrum shifts across the metasurface resonance, as shown in Figure 19c. A comparison of the third-harmonic signal from the silicon metasurface with the un-patterned silicon film shows a significant shift of the signal to shorter wavelengths for the metasurface, while it remains unchanged for the film (shown in Figure 19d). The photon acceleration efficiency for the third-harmonic signal was measured to be ~22%. The observed blue-shift was found to be in good agreement with a time varying photon mode amplitude model considering free carrier accumulation due to four-photon absorption process [112]. Photon acceleration based on time-varying optical processes in resonant dielectric metasurfaces presents a promising platform for performing robust pulse-shaping operations [112].

### 4.5. Higher Order Wave-Mixing Processes

**Figure 20:**
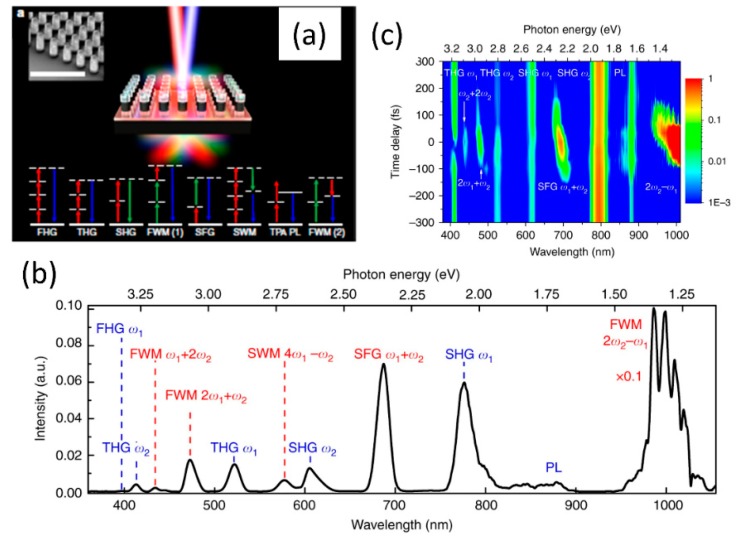
(**a**) Schematic of the Gallium Arsenide nanodisk array used for nonlinear wave mixing studies. (**b**) Experimentally measured spectra of various nonlinear wave mixing processes. The name of the various processes and their frequency relationship are labelled. (**c**) The dependence of the nonlinear wave-mixing spectra on the time-delay between the interacting excitation pulses. (Figures are reproduced with permission from ref. [113]).

The nonlinear optical studies are not restricted to just the second and third-order nonlinear optical processes. With high enough incident light fluence and high quality factor resonant metasurface medium with strong optical nonlinearities, various higher order processes greater than third order can also be observed. This is shown in Figure 20a for a Gallium Arsenide based meta-mixer consisting of nanodisk array [113]. Such structures support magnetic and electric dipolar resonances, as shown in the inset of Figure 20a. These resonances at the incident excitation laser wavelengths can be leveraged to study various nonlinear optical processes from second- and third-harmonic to four-wave mixing to fourth-harmonic generation and even six-wave mixing processes. With a high sensitivity spectrometer, the various nonlinear processes are spectrally resolved in Figure 20b. The dependence of the various nonlinear processes on time-delay of the interacting waves is also shown in Figure 20c. Even though the overall conversion efficiency is small, this is a promising direction towards realizing complex wave-mixing processes on a small footprint platform. Further enhancement in efficiency can be achieved by improving the quality factor of the resonances [77,78,79] or using such resonant structures in intra-cavity configuration [114].

### 4.6. Nonlinear Optics with Hybrid Metasurface

**Figure 21:**
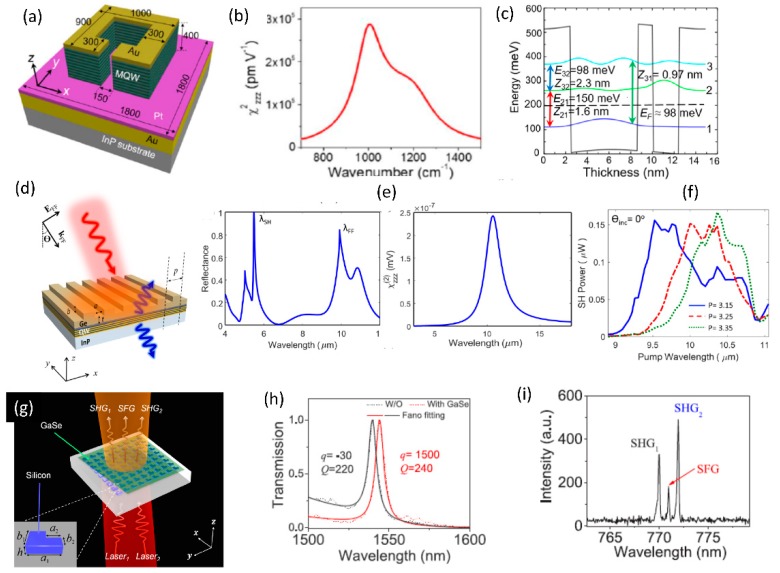
Various implementations of hybrid resonant metasurfaces for nonlinear optical applications. (**a**–**c**) Hybrid plasmonic-dielectric metasurfaces in which resonant nonlinearities in multi-quantum well structures are coupled with plasmonic resonances to study second harmonic generation. (**d**–**f**) All dielectric implementation of the hybrid resonant metasurface consisting of Germanium guided-mode resonance structures on top of the multi-quantum well structures. (**g**–**i**) Hybrid structures consisting of multi-layer Gallium Selenide on top of the asymmetric metasurface used for resonant enhancement of second-harmonic and sum-frequency generation. (Figures a–c are reproduced with permission from ref. [116], d–f are reproduced with permission from ref. [117], g–i are reproduced with permission from ref. [119]).

There is also interest in integration of dissimilar materials to realize hybrid metasurfaces with enhanced nonlinear optical properties when compared to the individual materials studied separately. Few examples of such hybrid integrated structures are shown in Figure 21. The hybrid integration of patterned metal metasurface with resonant quantum-well semiconductors has been studied with the objective of realizing doubly-resonant nonlinear optical metasurfaces [115,116]. The inherent resonant nonlinearities from the multi-quantum well structures are further amplified by the plasmonic resonant structures. Figure 21a shows the schematic of such hybrid plasmonic-dielectric structures. The corresponding resonant nonlinear susceptibility spectrum and the designed multi-quantum well structure is shown in Figure 21b and c respectively. The effective nonlinear susceptibility of the hybrid structure realized is close to 10^5^ pm/V, one of the highest nonlinear optical susceptibility reported for any solid-state material systems. The plasmonic structures have recently been replaced by dielectric metasurfaces, as shown in Figure 21d–f [117]. The structure consists of a one-dimensional germanium guided-mode resonant structure integrated on top of the multi-quantum layer stack. The designed optical resonances of the germanium guided-mode resonance structures and the associated resonant nonlinear optical susceptibility are shown in Figure 21e. 100s of nano-watt level second harmonic generation signal has been experimentally measured from these all-dielectric hybrid metasurfaces. Such structures with the fundamental wavelength in the mid infrared wavelength range are best suited for frequency up-conversion with high conversion efficiencies from wavelength regions where there is scarcity of high efficiency detectors to wavelength regions in the near-infrared and visible region where mature, high efficiency detectors are readily available.

There is also interest in integration of dielectric metasurfaces with two-dimensional layered materials in monolayer or multi-layer form. Two-dimensional materials offer robust nonlinear optical properties in terms of strength of the nonlinear optical susceptibility, its layer number and polarization dependence [118]. Such layered materials can be readily transferred to patterned dielectric structures with simple dry-transfer or chemical-vapor deposition techniques. Figure 21g–i shows one such hybrid integration of 2D material with silicon metasurface [119]. Multi-layer Gallium selenide dry-transferred onto asymmetric silicon resonant metasurface are utilized for resonant enhancement of second-harmonic and sum-frequency generation from the 2D material layer. A schematic of such structure is shown in Figure 21g, with a red-shift in the resonance spectrum in the presence of the 2D material shown in Figure 21h. The nonlinear optical process from the hybrid 2D material- dielectric metasurface is fairly strong that continuous-wave excitation has been used to generate second-harmonic and sum-frequency signals, as shown in the experimentally measured spectrum in Figure 21i. 

## 5. Concluding Remarks

In this review paper, an overview of various nonlinear optical processes studied in sub-wavelength periodic dielectric structures is presented. Dielectric structures are particularly attractive for nonlinear optical studies due to the ability to engineer field concentration to be located inside or outside the structure by design, high damage thresholds and ease of fabrication of complex structures. In particular, high refractive index dielectric structures are preferred for realizing such structures due to the high-quality factor of the resonances and the associated enhanced field strength. Resonant field enhancement mechanisms in such sub-wavelength structures are broadly classified here as guided-mode resonances and resonant metasurfaces. The physical mechanism behind the optical resonance phenomena in these structures are explained based on the guided-mode resonance phenomenon, EIT-like resonance and bound-state in continuum type resonances. The various nonlinear optical processes studied in these structures, which include second-/third-harmonic generation, four-wave mixing, sum-frequency generation, ultrafast optical switching based on Kerr and multiphoton absorption nonlinearities, photon acceleration of third harmonic generation signal, higher harmonic generation processes and hybrid resonant metasurfaces are discussed. Various dielectric structures in the form of one-dimensional gratings, two-dimensional arrays of nanodisks, bar-nanodisk structures, asymmetric bar dimers, asymmetric rectangular unit-cells, disordered nanodisk array, coupled GMR structures, heterogeneous structures are utilized for these nonlinear optical studies.

Looking ahead, such periodic dielectric structures are expected to find applications as miniaturized frequency converters in the form of intracavity photonic windows or as active fiber connectors [120,121]. The transition of this technology to practical, real world application does require improvements to certain aspects of this technology. In particular, scaling of the area of the metasurfaces, achieving higher conversion efficiencies, ability to extend to ultra-violet or infrared spectral windows with the exploration of novel structures and heterogenous integration capability will become essential. Optical metasurfaces are conventionally patterned using electron-beam lithography which renders it time consuming and difficult to scale to large area. The use of stepper lithography as used in standard electronic chip fabrication, interference lithography, imprint lithography or chemical synthesis techniques are promising to scale the metasurfaces to large areas [36,42]. The ability to combine multiple resonance phenomena such as quantum well based resonant nonlinearity with metasurface resonance is a promising direction to considerably improve the conversion efficiencies [116]. The use of high nonlinearity media on suitable low-index substrates, such as Gallium Phosphide on glass substrates [122] and high efficiency electro-optic polymers [123] are also possible direction to improve the efficiency. The use of multi-pass configuration, for example, in an intracavity application can also amplify the overall nonlinear process [120]. Furthermore, with emerging two-dimensional layered materials for photonic applications, the use of such materials in combination with dielectric metasurfaces is also seen as a promising direction [118]. The ability to switch or tune nonlinear optical functionality at high-speed will also be useful for fast optical modulation [124,125,126]. The exploration of high-quality factor resonances such as quasi bound-state in continuum is also an important direction to realize high efficiency resonant metasurfaces [83]. Overall, the emerging field of nonlinear optics in resonant metasurfaces and the resurgence it has given to guided-mode resonance platforms is creating lot of interest in nonlinear nanophotonics community. While practical applications are yet to emerge, the research efforts addressing this are in the right direction, which makes the future of this technology highly promising.
